# Optofluidic passive parity-time-symmetric systems

**DOI:** 10.1098/rsos.231200

**Published:** 2024-01-31

**Authors:** Franck Assogba Onanga, Hengky Chandrahalim

**Affiliations:** Department of Electrical and Computer Engineering, Air Force Institute of Technology, Wright-Patterson Air Force Base, OH 45433, USA

**Keywords:** parity-time symmetry, optofluidics, exceptional points, microsystems, optical waveguides

## Abstract

This research introduces a novel methodology of harnessing liquids to facilitate the realization of parity-time (*PT*)-symmetric optical waveguides on highly integrated microscale platforms. Additionally, we propose a realistic and detailed fabrication process flow, demonstrating the practical feasibility of fabricating our optofluidic system, thereby bridging the gap between theoretical design and actual implementation. Extensive research has been conducted over the past two decades on *PT*-symmetric systems across various fields, given their potential to foster a new generation of compact, power-efficient sensors and signal processors with enhanced performance. Passive *PT*-symmetry in optics can be achieved by evanescently coupling two optical waveguides and incorporating an optically lossy material into one of the waveguides. The essential coupling distance between two optical waveguides in air is usually less than 500 nm for near-infrared wavelengths and under 100 nm for ultraviolet wavelengths. This necessitates the construction of the coupling region via expensive and time-consuming electron beam lithography, posing a significant manufacturing challenge for the mass production of *PT*-symmetric optical systems. We propose a solution to this fabrication challenge by introducing liquids capable of dynamic flow between optical waveguides. This technique allows the attainment of evanescent wave coupling with coupling gap dimensions compatible with standard photolithography processes. Consequently, this paves the way for the cost-effective, rapid and large-scale production of *PT*-symmetric optofluidic systems, applicable across a wide range of fields.

## 1. Introduction

Over the past two decades, quantum physics has seen the advent and intensive exploration of a new theory. This theory asserts that a non-Hermitian Hamiltonian can represent physical systems, provided its energy spectra can be purely real within specific regimes, determined by the parameters of the Hamiltonian. Research has shown that the eigenenergy's reality is conditional on a particular type of symmetry within the non-Hermitian system. Such systems possess a combined reflection (parity) symmetry (*P*) and time-reversal symmetry (*T*), referred to as *PT*-symmetry [1,2].

This theory paves the way for unique features, particularly in open systems, where there may be gain and/or loss of particles or energy in interaction with the environment. Intriguingly, the Hamiltonian of a non-Hermitian *PT*-symmetric system provides a means to selectively induce complex eigenenergy for specific eigenstates [3]. As a result, a wealth of novel phenomena, absent in Hermitian systems, has been identified in non-Hermitian *PT*-symmetric systems in diverse fields such as electronic circuits [4–8], microwaves [9–11], acoustics [12,13] and optics [14–18].

Typically, the Hamiltonian, denoted as *H* = (*p*^2^/2*m*) + *V*(*y*) (where *p*^2^/2*m* and *V*(*y*) represent the kinetic and potential energy of the system, respectively), corresponds to a complex potential and satisfies the condition *V*(*y*) = V*( − *y*). However, when the non-Hermiticity parameter (variable in complex potential) surpasses a certain threshold, a portion of the Hamiltonian's energy spectrum ceases to be real. This threshold corresponds with the emergence of an exceptional point (*EP*)—special degeneracies characterized by a coalescence of not just the eigenvalues, but also the corresponding eigenvectors of the system [1,19].

The concept of *PT*-symmetry, as understood in the framework of the single-particle Schrödinger equation in quantum mechanics, can be appropriately translated to the realm of Maxwell's electromagnetics. Pursuing this analogy, we consider an electromagnetic medium characterized by a complex refractive index, $n(y)=\sqrt{\varepsilon (y)}>0$, where ε(*y*) is the permittivity and *n*(*y*) is assumed to be positive. For cases where *n*(*y*) varies slowly, a parallel can be drawn between the Schrödinger equation and the Maxwell wave equation. This analogy is established by replacing the temporal evolution in quantum mechanics with spatial propagation along the *x*-dimension in optics and introducing a complex potential *V*(*y*) = *k*_0_*n*(*y*), where *k*_0_ is the wavenumber. Consequently, the complex potential *V*(*y*) correlates directly with the refractive index *n*(*y*) of the medium. Under this framework, *PT*-symmetry in optics is achieved when the condition *n*(*y*) = n*( − *y*) is satisfied, mirroring the *PT*-symmetry requirements in quantum mechanics [14,19].

In optics, gain via stimulated emission and loss through absorption media are naturally occurring phenomena, allowing the implementation of *PT*-symmetric optical systems as periodic constructs comprising balanced amplifying (gain) and absorbing (loss) media with balanced rates of ± *γ*, resulting in a system's complex refractive index distribution corresponding to *n*(*y*). Such systems, which may include coupled resonators, coupled waveguides, modulating waveguides with deposited metal on part of a single waveguide, and fibre ring resonator systems, exhibit numerous non-trivial phenomena stemming from the *EP*s. These phenomena have been thoroughly explored due to their intriguing properties.

Despite the abundance of novel findings associated with *PT*-symmetric systems, their design and physical realization pose significant challenges, particularly when it comes to tuning system coupling. Experimentalists often make monumental efforts to reach critical coupling or to precisely dice chips and couple the system at the edge on optical table set-ups, using bulky and expensive translational stages to create multiple samples needed to study behaviour across an *EP* [20,21].

**Figure 1.**
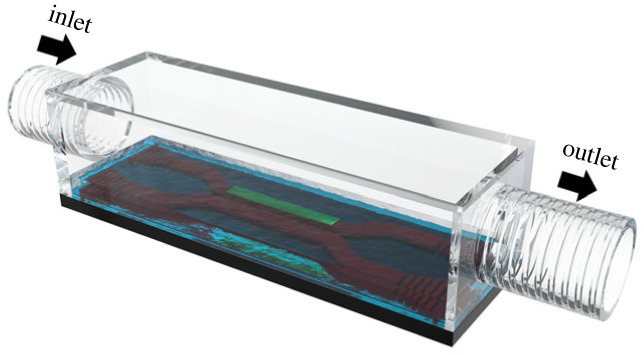
Illustration of an optofluidic microsystem, featuring two optical waveguides coupled with a liquid, the refractive index of which can be dynamically adjusted in real time by introducing liquids of varying refractive indices from the inlet to the outlet. A lossy material is patterned on one of the waveguides. The dynamic adjustment of the liquid's refractive index facilitates the modification of the coupling strength between the two waveguides, thereby enabling operation within the *PT*-symmetric, *PT*-broken-symmetric and exceptional point regimes. This illustration serves to elucidate the functionality of the optofluidic system by vividly detailing its constituent subcomponents. It should be noted that the actual scale and physical form of the system may differ substantially from the image depicted here.

Optofluidic microsystems, where microscale optical elements and microfluidic frameworks merge on a shared substrate as illustrated in figure 1, provide a solution for this issue. These systems offer ease in tuning and managing the light coupling between proximate optical elements on a shared substrate [22–25], thereby constituting an excellent platform for fluid-sensing applications when operating around an *EP*. Additionally, these systems enable the parallel fabrication of various *PT*-symmetric systems via standard photolithography processes. Crucially, the refractive index of the liquid-filled coupling gaps can be adjusted in real time to comply with the constraints of standard UV-based photolithography [23]. The current study theoretically demonstrates the ability of an optofluidic microsystem to modulate a coupled optical waveguide system, thereby enabling operation within *PT*-symmetric, *PT*-broken-symmetric and *EP* regimes. A pragmatic fabrication process flow for creating the proposed optofluidic microsystem is outlined, and the theoretical proposition is corroborated through comprehensive finite-element modelling.

## 2. Methods

Numerous studies have established that optics serves as an ideal domain for exploring the fundamentals of *PT*-symmetry, both from theoretical and experimental perspectives. A mathematical analogy between the Schrödinger equation and the electromagnetic wave equation in the paraxial limit streamlines the incorporation of *PT*-symmetry into optical fields. Here, non-Hermiticity can be engineered from the complex refractive index of the system materials [15,26]. Under such circumstances, light dynamics, characterized by a slowly varying envelope of the electric field, *E*, with negligible transverse diffraction angles, can be represented as follows:

2.1 $$i\frac{\partial E}{\partial x}+\frac{1}{2k_{0}}\frac{\partial^{2}E}{\partial y^{2}}+k_{0}[n_{R}(y)+in_{I}(y)]E=0.$$

In equation (2.1), *k*_0_ is defined as 2*π*/*λ*_0_, where *λ*_0_ signifies the vacuum wavelength of light, and *k* = *k*_0_*n*_0_. These definitions are predicated on the assumption that the complex refractive index distribution can be represented as *n*(*y*) = *n*_0_ + *n_R_*(*y*) + *in_I_*(*y*), wherein *n*_0_ constitutes a real background refractive index, *n_R_*(*y*) defines the real index profile of the structure, and *in_I_*(*y*) symbolizes the gain or loss component. Furthermore, it is considered that *n*_0_ significantly outweighs *n_R_* and *n_I_*. In this context, *n*(*y*) assumes the role of a complex optical potential that satisfies the conditions *n_R_*(*y*) = *n_R_*( − *y*) and *n_I_*(*y*) = −*n_I_*( − *y*). In our passive optofluidic configuration, the *PT*-symmetry condition is applied along the *y*-axis, which aligns with the width of the waveguides. This means that for any point ‘*y*’ along this axis, the real part of the refractive index is mirrored at ‘−*y*,’ while the imaginary part has an inverse symmetry.

The phenomenon of *PT*-symmetry breaking in finite-dimensional systems, characterized by a transition from an entirely real energy spectrum to a complex one, is typically associated with the emergence of *EP*s. A key distinction between exceptional points and diabolic points (Hermitian degeneracies), denoted as *DP*, is their respective sensitivity to perturbations. Specifically, a minor perturbation ε (where *ε* ≪ 1) around the *EP*s of a system results in a frequency shift on the order of $\varepsilon^{1/m}$. Here, *m* represents the number of coalescing eigenvalues, thereby defining the order of the *EP*. This contrasts with a Hermitian system where the same perturbation causes a frequency shift that scales linearly with ε. The singularity of *EP*s has been leveraged to design high-sensitivity detection devices, including on-chip cavity sensors [3,20,21].

The frequency shift near exceptional points, induced by minor perturbations, is not merely a theoretical nuance but has profound practical implications in our study. This shift is intrinsically linked to the phenomenon of optical mode splitting, a critical factor in *PT*-symmetric systems. Mode splitting, the process where a single resonant mode diverges into multiple distinct modes each with its own frequency, becomes especially pronounced near *EP*s due to the system's heightened sensitivity. In *PT*-symmetric optofluidic systems, this splitting can be triggered by variations in fluid properties or alterations in the waveguide structure, leading to a significant shift in the resonant frequencies of the modes.

Exceptional points are specific conditions within the parameter space of our *PT*-symmetric system where the eigenvalues of the system's Hamiltonian coalesce. Unlike typical degeneracies in Hermitian systems (diabolic points), at an *EP*, not only do the eigenvalues converge, but their corresponding eigenvectors also merge. This convergence results in a unique phenomenon where the system loses its usual full rank, and the eigenvectors become self-orthogonal. The physical manifestation of this mathematical property in optofluidic systems is a point of transition between distinctly different operational modes—from a *PT*-symmetric phase to a *PT*-broken phase.

In our specific study, the *EP*s emerge as a consequence of the interplay between the loss in one of the coupled waveguides and the evanescent coupling between the waveguides. At these points, the behaviour of light in the coupled waveguide system undergoes a fundamental shift. Below the threshold (where the loss is smaller than the coupling strength), the system behaves in a *PT*-symmetric manner with real eigenvalues, indicating stable propagation of light. At the *EP*, these eigenvalues become degenerate, signifying a critical point of transition.

Furthermore, the degeneracy of eigenvectors at *EP*s in our system plays a significant role in defining the operational characteristics. At an *EP*, due to the collapse of eigenvectors into a single state, the system exhibits a heightened sensitivity to external perturbations. This sensitivity is a cornerstone of the potential applications we explore, such as highly responsive optical sensors. The modes of the waveguide system, under these conditions, exhibit unique interference and intensity distribution patterns, a direct consequence of the eigenvector degeneracy.

From a theoretical perspective, we consider the following 2 **×** 2 Hamiltonian matrix, denoted as *H*_PT_, that represents a system of two coupled optical waveguides. In this scenario, one of the waveguides undergoes a loss while the other experiences a gain.

2.2 $$H_{\mathrm{PT}}=\left[\begin{array}{cc} \omega_{0}+i\alpha & \mu \\ \mu & \omega_{0}-i\alpha \end{array}\right].$$

In equation (2.2), *ω*_0_ symbolizes the resonant frequency in the absence of gain and loss, while *μ* and *α* denote the coupling strength between the two waveguides and the gain/loss strength within each waveguide, respectively.

To bridge the theoretical description of *PT*-symmetry in optical fields with our specific model of coupled waveguides, we consider the influence of the refractive index profile on the system dynamics. The term *k*_0_[*n_R_*(*y*) + *in_I_*(*y*)] in equation (2.1) encapsulates the refractive index's impact on light propagation. In the scenario of two coupled waveguides, the parameter *ω*_0_ in equation (2.2) corresponds to the resonant frequency influenced by the background refractive index *n*_0_​. The loss parameter *α* is directly related to the imaginary part of the refractive index *n_I_*(*y*), denoting the attenuation rate in the waveguides. The coupling strength *μ* is affected by the overlap of the evanescent fields, linked to the real part of the refractive index *n_R_*(*y*) and the waveguides' geometry.

The Hamiltonian *H*_PT_, although non-Hermitian, maintains *PT*-symmetry provided the gain/loss strength is non-zero. In this scenario, the parity operator *P* is denoted by the Pauli matrix $\sigma_{x}=\left[\begin{array}{cc} 0 & 1\\ 1 & 0 \end{array}\right]$, while the time reversal operator *T* is represented by the complex conjugate. The eigenenergies of the system are defined by the expression $E_{PT\mp }=\omega_{0}\mp \sqrt{\mu^{2}-\alpha^{2}}$. When *α* < *μ*, indicative of a weaker gain and loss dynamic within the waveguides relative to their coupling, the eigenenergies are entirely real and distinct, positioning the system within the *PT*-symmetric phase. At the symmetry-breaking transition point, the gain/loss strength parameter perfectly counterbalances the coupling strength (*α* = *μ*), causing the eigenenergies and their corresponding eigenvectors to coalesce into an exceptional point. By contrast, when *α* > *μ*, the system transitions into the *PT*-broken phase, characterized by an amplified mode in one waveguide and a decaying mode in the other.

Equation (2.2) describes a balanced *PT*-symmetric system in a directional coupler, an experimental realization of which is complex, as it necessitates the integration of an active gain medium in one of the optical waveguides. To mitigate these challenges, passive *PT*-symmetric settings defined by imbalanced losses, or neutral-loss coupled systems, have emerged as prevalent strategies for the experimental execution of *PT*-symmetric systems. Consider, for instance, a directional coupler system comprising two identical waveguides, into one of which a methodical and substantial amount of loss, *γ* = 2*α*, is introduced. Adopting the Schrödinger equation from quantum mechanics, the propagation of a light beam within such a structure can be articulated by the following equation:

2.3 $$i\frac{d}{dt}\left[\begin{array}{c} E_{1}\\ E_{2} \end{array}\right]=H_{\mathrm{NL}}\left[\begin{array}{c} E_{1}\\ E_{2} \end{array}\right],$$

where the Hamiltonian of the coupled neutral/loss system, $H_{\mathrm{NL}}=\left[\begin{array}{cc} \omega_{0} & \mu \\ \mu & \omega_{0}-2i\alpha \end{array}\right]$

Applying the gauge transformation $\left[\begin{array}{c} E_{1}\\ E_{2} \end{array}\right]=e^{-\alpha t}\left[\begin{array}{c} E_{1}^{\prime }\\ E_{2}^{\prime } \end{array}\right]$, yields

2.4 $$\begin{array}{rl} i\frac{d}{dt}\left[\begin{array}{c} E_{1}^{\prime }\\ E_{2}^{\prime } \end{array}\right] & =i\alpha \left[\begin{array}{c} E_{1}^{\prime }\\ E_{2}^{\prime } \end{array}\right]+\left[\begin{array}{cc} \omega_{0} & \mu \\ \mu & \omega_{0}-2i\alpha \end{array}\right]\left[\begin{array}{c} E_{1}^{\prime }\\ E_{2}^{\prime } \end{array}\right]\\ & =\left[\begin{array}{cc} \omega_{0}+i\alpha & \mu \\ \mu & \omega_{0}-i\alpha \end{array}\right]\left[\begin{array}{c} E_{1}^{\prime }\\ E_{2}^{\prime } \end{array}\right]=H_{\mathrm{PT}}\left[\begin{array}{c} E_{1}^{\prime }\\ E_{2}^{\prime } \end{array}\right]. \end{array}$$

Thus, the Hamiltonian of the coupled neutral/loss system, *H*_NL_ can be written as

2.5 $$\begin{array}{rl} H_{\mathrm{NL}} & =\left[\begin{array}{cc} \omega_{0} & \mu \\ \mu & \omega_{0}-2i\alpha \end{array}\right]=\left[\begin{array}{cc} -i\alpha & 0\\ 0 & -i\alpha \end{array}\right]\\ & \;+\left[\begin{array}{cc} \omega_{0}+i\alpha & \mu \\ \mu & \omega_{0}-i\alpha \end{array}\right]=H_{L}+H_{\mathrm{PT}}, \end{array}$$

where the Hamiltonian of the lossy uncoupled system, $H_{L}=\left[\begin{array}{cc} -i\alpha & 0\\ 0 & -i\alpha \end{array}\right]$. Equation (2.5) encapsulates the physical implication that the dynamics of a neutral-loss coupled waveguides system mirror those of a balanced gain-loss coupled waveguides system, apart from a global exponential damping or amplification. The dynamics of the neutral-loss coupled waveguides system can also be described by the eigenenergies of the passive neutral-loss system, as expressed by $E_{\mathrm{NL}\mp }=\omega_{0}-i\alpha \mp \sqrt{\mu^{2}-\alpha^{2}}=E_{\mathrm{PT}\mp }-i\alpha$. Here, *E*_NL_ and *E*_PT_ denote the eigenenergies of the passive neutral-loss system and the balanced gain-loss system, respectively.

Fabricated using standard complementary metal-oxide semiconductor (CMOS) technologies, chip-scale optical waveguide arrays facilitate the creation of low-cost, ultra-compact and highly sensitive microscale sensors. These sensors are widely employed in an array of applications, spanning biological, chemical and environmental detections. Integrated photonics platforms provide solutions to the demanding requirements for compact and affordable sensors. Indeed, integrated photonics has been a pivotal technology enabler, facilitating the construction of complex optical components for an abundance of applications, including communications, high-performance computing and on-chip sensing. In particular, silicon nitride (Si_3_N_4_) boasts exceptional optical, chemical and mechanical properties, such as low index contrast, superior fabrication tolerance, reduced insertion loss and thermal stability. Consequently, Si_3_N_4_ has been a broadly researched platform upon which a myriad of active and passive devices have been implemented to date [27,28].

**Figure 2.**
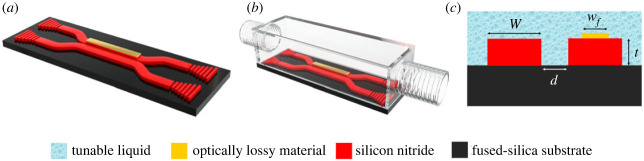
Schematic diagrams of (*a*) optical waveguides situated on a fused-silica substrate, featuring input and output gratings as well as an optically lossy material on one of the waveguides, (*b*) an optofluidic waveguide system encapsulated within a transparent fluidic host equipped with inlet and outlet channels, hosting the waveguide system depicted in figure 2*a*, and (*c*) a cross-sectional view of the two waveguides with fluidic cladding, supplemented with annotations to elucidate the symbols of the key dimensions. These schematics are intended to vividly detail the constituent subcomponents of the optofluidic system. Please note that the actual scale and physical form of the system may deviate substantially from the schematics presented here.

This study presents the design, fabrication process and finite-element modelling of an optofluidic, passive *PT*-symmetric system with potential applications in light management devices such as switches, light modulators and optical sensors. The integrated optofluidic microsystem consists of two nearly identical, coupled silicon nitride waveguides. Each waveguide exhibits cross-sectional dimensions with a width, *W*, of 2 µm and a thickness, *t*, of 1 µm, optimized to support single-mode operation at an approximate wavelength of 1550 nm, as illustrated in figure 2*a*. While one of the waveguides remains neutral, the other is overlaid with a thin layer of an optically lossy material. In this study, we use a 100 nm-thick layer of copper as the lossy material due to its wide availability and ease of deposition using standard semiconductor processes. The waveguides are separated by a coupling gap, denoted by *d*. The coupling strength between the two waveguides can be dynamically modulated by introducing a transparent liquid with a refractive index slightly lower than that of the waveguides. As illustrated in figure 2*b*, the proposed optofluidic system is fabricated on a fused-silica substrate and features a transparent fluidic host with inlet and outlet. Figure 2*c* shows a cross-sectional view of the coupled waveguides on a fused-silica substrate with liquid cladding.

In the design of our optofluidic system, the selection of fused silica as the host material was a strategic decision, underpinned by several of its advantageous properties. Fused silica is renowned for its broad transparency to light from 300 to 2000 nm, an attribute essential for optical applications. This wide spectral transparency range facilitates efficient light propagation, making it an ideal candidate for on-chip optical systems. Notably, at wavelengths of 500 and 1550 nm, the refractive indices of fused silica are approximately 1.460 and 1.444, respectively, which are favourable for a wide range of optical applications. Furthermore, its mechanical robustness ensures durability and long-term stability under various operational conditions. The chemical inertness of fused silica is another critical factor, as it allows for a wide range of real-world applications, particularly in on-chip chemical and biological sensing, where resistance to corrosive substances and biological materials is paramount.

While fused silica inherently lacks optical tunability, this characteristic actually presents a distinct advantage in our optofluidic system design. Its stable optical properties, including a consistent refractive index at different wavelengths, provide a reliable and predictable platform for precise optical manipulations. This stability ensures that any optical tuning or modulation required can be externally controlled and implemented, for instance, through the manipulation of the surrounding optofluidic environment or the integration of tunable optical components. Thus, the non-tunability of fused silica contributes to the overall robustness and reliability of our optofluidic system, making it a suitable material for applications requiring stable and precise optical performance.

Moreover, the compatibility of fused silica with standard semiconductor fabrication techniques significantly influenced our choice. Its amenability to conventional processes such as lithography and etching, commonly used in semiconductor manufacturing, facilitates ease of fabrication. This compatibility not only streamlines the production process but also reduces the cost and complexity involved in the development of optofluidic devices. Therefore, the integration of fused silica into our optofluidic system aligns with our objective of developing a robust, efficient and versatile platform for advanced optical applications.

It is important to note that the coupling distance between two optical waveguides in air is typically less than 500 nm for near-infrared wavelengths and under 100 nm for ultraviolet wavelengths. Such small geometries often necessitate the use of electron beam lithography, a process that is expensive, slow and labour-intensive. However, the proposed optofluidic system enables evanescent coupling between waveguides with a coupling gap larger than 1.5 µm, which can be conveniently patterned using a standard UV lithography system. Input light to the optical grating and output light from the optical grating are coupled via free space optics using lenses. The proposed optofluidic system lays the groundwork for the cost-effective, rapid and large-scale production of *PT*-symmetric optofluidic systems, demonstrating significant potential for application across diverse fields.

**Figure 3.**
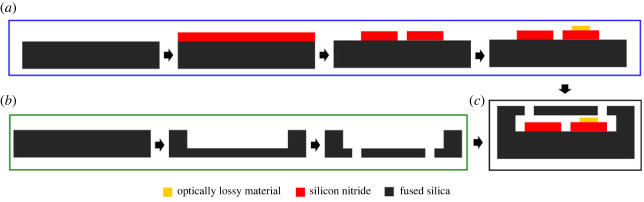
Schematic diagrams describing the fabrication process flow for the optofluidic waveguides system. (*a*) Details the fabrication process flow for the coupled waveguides on a fused-silica substrate. (*b*) Outlines the fabrication process flow for the fluidic host. (*c*) Shows a schematic of the fully integrated optofluidic system, achieved by bonding the fabricated fused-silica substrate bearing the optical waveguides with the fluidic host.

The fabrication process of the optofluidic waveguide system involves bonding two separately fabricated fused-silica substrates, as depicted in figure 3. The procedure for creating the optical waveguides on the first fused-silica substrate is detailed in figure 3*a*. The process commences with the deposition of a 1 µm thick layer of silicon nitride film on a 4″ fused-silica wafer using a low-pressure chemical vapour deposition (LPCVD) method, which ensures reliable reproduction and is suitable for volume production [29–31]. Subsequently, the SPR 220-3 positive tone photoresist is patterned with waveguide structures utilizing a SUSS MA/BA-6 contact aligner. Reactive ion etching (RIE) is then employed to transfer the waveguide patterns into the silicon nitride layer. CF_4_ and H_2_ gases, with flow rates of 40 and 3 standard cubic centimetres per minute (sccm), respectively, are used in the RIE process to achieve an anisotropic profile with optically smooth sidewalls [32]. A thin film of an optically lossy material is patterned onto one of the waveguides using a metal lift-off process. In this study, copper is selected as the lossy material due to its widespread availability and the simplicity of its deposition via a standard metal evaporation process. The microfluidic encapsulation layer is fabricated using a second 4″ fused-silica wafer, as shown in figure 3*b*. The SPR 220-3 photoresist is patterned with fluidic channels using a SUSS MA/BA-6 contact aligner. A 49% hydrofluoric acid (HF) solution is then used to wet-etch 5 µm deep fluidic channels into the fused-silica wafer [31]. Subsequently, the wafer is spin-coated with a negative-tone photoresist (KMPR), and a contact aligner is used for UV light exposure. A reactive ion glass etcher is then employed to etch inlets and outlets through the fused-silica wafer [33–35]. Finally, the first fused-silica wafer with optical waveguides is bonded to the second fused-silica wafer with fluidic channels using a low-temperature glass bonding process [36].

## 3. Results and discussion

In this study, we employ the effective index method (EIM) [37] to a sizable cross-section of a rectangular optical waveguide. The objective is to approximate the optimal height and width of a silicon nitride waveguide core situated on a fused-silica substrate, such that it can maintain single-mode operation at an operating wavelength, *λ*_0_, of 1550 nm. The EIM is a suitable approximation when the waveguide width is larger than its thickness, i.e. *W* > *t* [38]. As per the EIM, the cross-sectional dimensions of the nitride waveguide should feature a width, *W*, of 2 µm and a thickness, *t*, of 1 µm.

After establishing the silicon nitride waveguide dimensions that support single-mode operation at telecommunication wavelength, we analyse the coupling strength between the two waveguides as a function of the coupling gap. A coupling gap range of 1 to 2 µm is selected, which can conveniently be patterned using standard contact lithography. The theoretical calculation of the coupling strength between two waveguides can be accomplished using equation (3.1), as detailed in [38],

3.1 $$\mu =\frac{2}{\beta d_{E}}\frac{h^{2}\delta }{h^{2}+\delta^{2}}e^{-\delta d},$$

where $\delta^{2}=\beta^{2}-n_{cl}^{2}k_{0}^{2}$, $h^{2}=n_{c}^{2}k_{0}^{2}-\beta^{2}$, *k*_0_ = 2*π*/*λ*_0_, *β* denotes the propagation constant, and *d_E_* is the effective width of the waveguide.

**Figure 4.**
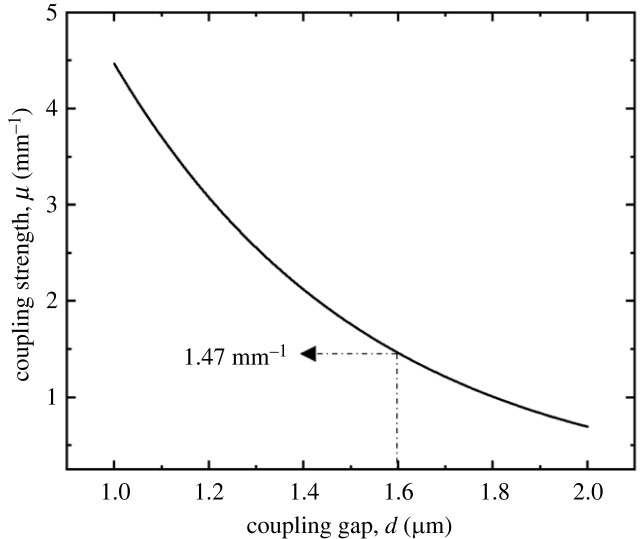
A graph of analytically calculated coupling strength, *μ*, between the fundamental modes of the two waveguides presented as a function of the coupling gap, *d*. In this analysis, an experimental liquid cladding with a refractive index that is 4% lower than the silicon nitride core refractive index is assumed.

The analytically calculated coupling strength between the fundamental modes of the two waveguides, as a function of the coupling gap, is graphically plotted in figure 4. For our coupled waveguide design, we adopt a coupling gap of 1.6 µm, as this feature size can be effortlessly patterned using a standard UV photolithography process. With *d* = 1.6 µm, the corresponding coupling strength is 1.47 mm^−1^.

**Figure 5.**
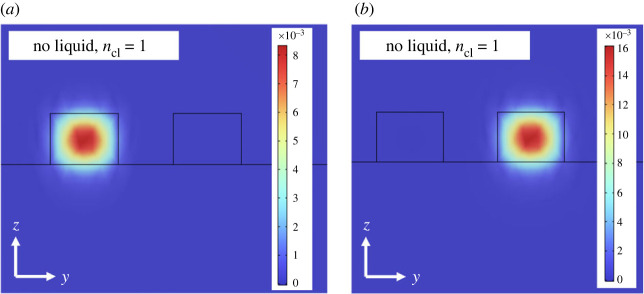
Finite-element simulation result of coupled silicon nitride waveguides, each having a thickness of 1 µm and a width of 2 µm, separated by 1.6 µm on a fused-silica substrate. The analysis includes (*a*) the symmetric y-polarized mode and (*b*) the anti-symmetric y-polarized mode. The model incorporates air as the optical cladding material. The simulation shows no evidence of evanescent coupling between the two nitride waveguides.

The objective of this work is to enable the dynamic tuning of *PT*-symmetric systems that can be mass-produced in parallel using a conventional lithography process. To achieve this, the coupling gap between the two waveguides should be larger than 1 µm. We have modelled the coupling dynamics of two parallel silicon nitride waveguides, separated by 1.6 µm, on a fused-silica substrate. The model was developed utilizing the finite-element method within the COMSOL Multiphysics software package, taking into account the precise material properties and boundary conditions pertinent to chip-scale optical waveguides. Given the proportions of our system—where the propagation length considerably exceeds the wavelength—the Electromagnetic Waves, Beam Envelopes interface proves to be particularly appropriate. This is because the mesh need not resolve the wave on a wavelength scale, but rather the beating between the two waves. The domain size was defined as 12 by 10 µm in the y and z directions, respectively. Two numeric ports were used for each input and output boundary, defining the lowest symmetric and antisymmetric modes of the waveguide structure. Our initial simulation considered the two waveguides without the incorporation of liquid cladding (cladding refractive index, *n_cl_* = 1). As depicted by the finite-element simulation results presented in figure 5, no evanescent coupling is observed between the two waveguides.

**Figure 6.**
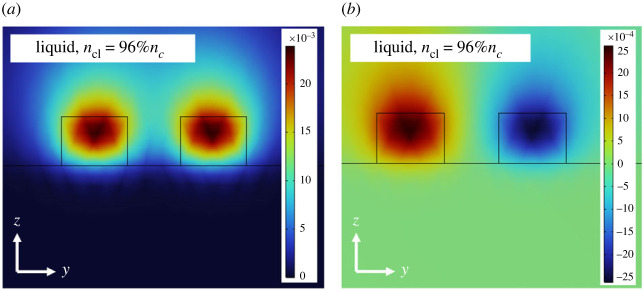
Finite-element simulation result illustrating the coupled silicon nitride waveguides, each with a thickness of 1 µm and a width of 2 µm, separated by 1.6 µm on a fused-silica substrate. The optical cladding material in this model is an experimental liquid whose refractive index, *n_cl_* is 4% lower than that of silicon nitride (*n_c_*). The analysis includes (*a*) the symmetric y-polarized mode and (*b*) the anti-symmetric y-polarized mode. The simulation provides evidence of evanescent coupling between the two nitride waveguides, facilitated by the high-refractive-index liquid cladding.

By leveraging the availability of high-refractive-index fluids [39,40], we can dynamically modulate the waveguide's surroundings to achieve a refractive index, *n_cl_*, sufficiently close to that of the waveguide core, *n_c_*. Such an approach significantly enhances the evanescent coupling between the two waveguides. As a proof of concept, we selected an experimental liquid cladding with *n_cl_* that is 4% lower than *n_c_* to investigate the coupling behaviour of two waveguides using COMSOL Multiphysics. The simulation results, as presented in figure 6, provide clear evidence of evanescent coupling between the two nitride waveguides, facilitated by the high-refractive-index liquid cladding.

In the present work, we employ a design framework that involves the coupling of two identical waveguides, one of which is engineered to incorporate optically lossy characteristics. Notably, this architecture does not necessitate the incorporation of optically active gain materials, thus simplifying the overall fabrication process and system complexity. For the system to operate within the distinct realms of *PT*-symmetric, *PT*-broken-symmetric and exceptional point regimes, a critical parameter that needs precise modulation is the coupling strength, *μ*, between the two waveguides.

**Figure 7.**
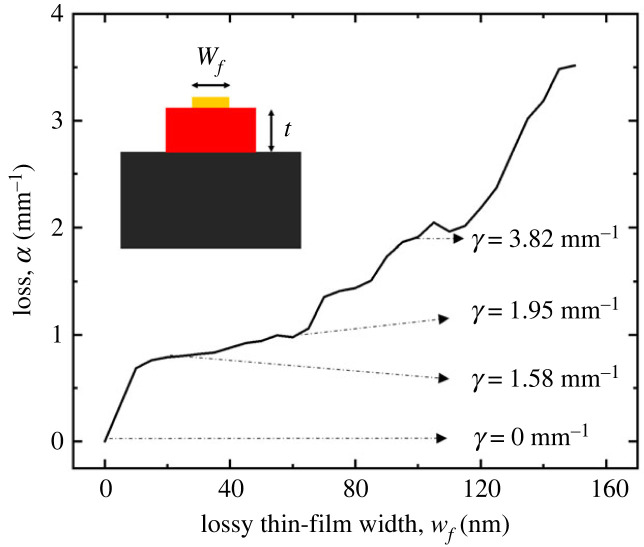
Optical loss, *γ* = 2*α*, in the waveguide containing an optically lossy material (shown in the inset image), represented as a function of the width, *w_f_*, of the lossy material. This relationship is simulated using a finite-element software package over one coupling length.

In addition, the degree of loss, *γ*, within the lossy waveguide must also be meticulously controlled to balance *μ*. Through the careful adjustment of these key parameters, we can achieve a robust operational range across *PT*-symmetric, *PT*-broken-symmetric and exceptional point regimes, thereby laying the foundation for innovative advancements in the field of optofluidic passive *PT*-symmetry in optics. In this study, we have selected a 100 nm-thick layer of copper, having a width denoted by *w_f_*, as the lossy material of choice, as shown in the inset of figure 7. This selection is grounded in copper's widespread availability and the simplicity of its deposition through a standard metal evaporation process. The graph presented in figure 7 delineates the relationship between *γ* within a waveguide containing an optically lossy material, and *w_f_*. This curve has been derived through careful simulations using COMSOL Multiphysics®, thereby capturing the intricate interplay between *w_f_* and *γ*. This analysis provides a profound understanding of the design considerations essential for controlling the parameter *γ*, allowing it to be less than, greater than, or equal to 2*μ* within a passive optofluidic waveguide system.

**Figure 8.**
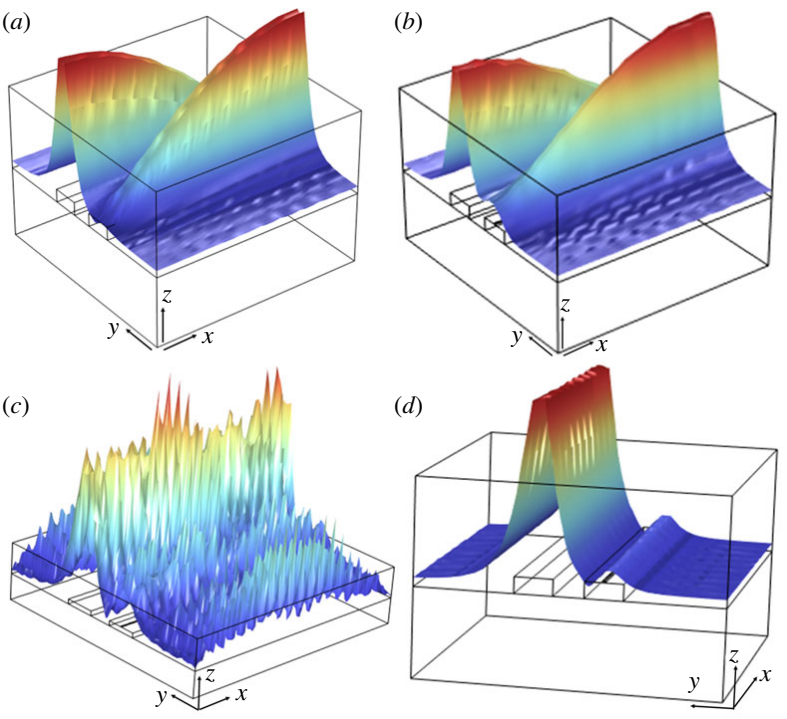
Finite-element analysis of a coupled waveguide system, showcasing a fluidic cladding with a refractive index 4% lower than that of the core waveguides, and incorporating a coupling gap of 1.6 µm (corresponding to a coupling strength of *μ* = 1.47 mm^−1^). The system illustrates different optical loss scenarios within one of the waveguides, as characterized by: (*a*) *w_f_* = 0 or *γ* = 0, (*b*) *w_f_* = 20 nm or *γ* = 1.58 mm^−1^, (*c*) *w_f_* = 60 nm or *γ* = 1.95 mm^−1^, and (*d*) *w_f_* = 100 nm or *γ* = 3.82 mm^−1^. Note: The dimensions of the structures presented in the finite-element simulation results are not to scale.

In the subsequent phase of this study, COMSOL Multiphysics was employed to simulate coupled waveguides, with one (the right waveguide) exhibiting a varying degree of optical loss, as demonstrated in figure 8. Within the context of this investigation, the separation between the waveguides was held constant at 1.6 µm, and the refractive index of the liquid optical cladding was configured to be 4% lower than the silicon nitride waveguide refractive index. The simulation model employed two numeric ports for each input and output boundary, thereby defining the fundamental symmetric and antisymmetric modes of the waveguide structure [41]. The neutral waveguide (i.e. the waveguide without loss) was selectively excited by superposing the lowest symmetric and antisymmetric y-polarized modes, with corresponding propagation constants *β*_1_ = 7.8425 µm and *β*_2_ = 7.8324 µm, through the designated input numeric ports. This specific set-up was designed to isolate and remove the rapid phase variation synchronizing with the first mode. Consequently, the total electric field, denoted by *E*(*x*), can be mathematically expressed as the summation of the electric fields of the two modes, $E(x)=E_{1}e^{\left(-j\beta_{1}x\right)}+E_{2}e^{\left(-j\beta_{2}x\right)}=[E_{1}+E_{2}e^{\left(-j(\beta_{2}-\beta_{1})x\right)}]e^{\left(-j\beta_{1}x\right)}$ and the coupling length, *L*_C_ is defined by *L*_C_ = 2*π*/*β*_2_ − *β*_1_ [41].

In the case of a conventional Hermitian system, where no lossy material is added to one of the waveguides, or where the optical loss parameter *γ* = 0, the electric field is observed to increase in the receiving waveguide and decrease in the exciting waveguide, as depicted in figure 8*a*. As a 20 nm-wide copper film is introduced to one of the waveguides, generating an optical loss of *γ* = 1.58 mm^−1^, the system transitions to a non-Hermitian state, yet still manifests Hermitian-like behaviour, as evidenced in figure 8*b*. With an increase in the copper film's width to 60 nm, the optical loss amplifies to *γ* = 1.95 mm^−1^, leading to the observation of distorted, leaky optical modes within the system, as shown in figure 8*c*. This stage is indicative of the system's approach toward the *PT*-symmetry threshold, or the exceptional point. Further escalation in the copper film's width to 100 nm causes the optical loss to reach *γ* = 3.82 mm^−1^, corresponding to *α* = *γ*/2 = 1.91 mm^−1^. This value exceeds the coupling strength (*μ*) of 1.47 mm^−1^ for the predefined coupling gap of 1.6 µm. This results in the system attaining a *PT*-broken-symmetry state.

We have observed a complex interplay of evanescent coupling dynamics as the optical loss is incrementally increased in one of the waveguides. The erratic coupling behaviour between the two waveguides is presented in figure 8*c*. This system, inherently non-Hermitian due to the asymmetric loss distribution, approaches the *PT*-symmetry threshold, a critical point where the balance between neutral and loss is precisely maintained. As the system nears this threshold, the optical mode exhibits rapid tunnelling between the two waveguides, leading to a chaotic coupling dynamic. This behaviour can be attributed to the heightened sensitivity of the system's eigenvalues to the loss parameter, as it verges on the exceptional point.

Upon surpassing the exceptional point, with further augmentation of optically lossy material in the lossy waveguide, the system transitions into the *PT*-broken-symmetry regime. In this regime, the non-Hermitian nature of the system becomes pronounced, leading to a distinct alteration in the eigenvalue spectrum. This transition is marked by a qualitative change in the optical coupling behaviour, wherein the previously observed erratic coupling dynamics stabilize, reflecting a fundamental shift in the system's underlying physical properties. This phenomenon can be observed in figure 8*d*.

While various geometries of optically lossy materials can be designed to operate within *PT*-symmetric, *PT*-broken-symmetric, and exceptional point regimes, our optofluidic platform uniquely enables the real-time tuning of the liquid cladding's refractive index to function across different *PT* phases. The application of this approach requires careful identification of the loss geometry that prompts the optofluidic system to function at the exceptional point, where the coupling strength equals half of the optical loss (*μ* = *γ*/2 = *α*). This consideration is vital, as it allows the geometry of the optically lossy material to be designed for operation at the exceptional point while dynamically varying the refractive index of the liquid cladding to operate within *PT*-symmetric, *PT*-broken-symmetric, or exceptional point regimes, as desired.

The plot in figure 7 confirms that the optical loss induced by thin optically lossy material increases proportionally with the width of the material. Previous studies have reported that the total optical transmission within the system decreases during the *PT*-symmetry phase, reaching a minimum at the exceptional point, before subsequently increasing as the system enters the *PT*-broken-symmetry phase [42].

**Figure 9.**
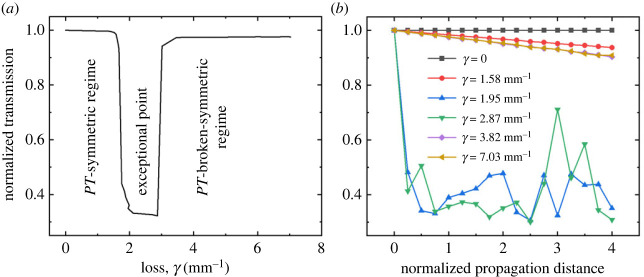
(*a*) The normalized total optical transmission of the coupled waveguide system as a function of optical loss, elucidating distinct *PT* phases characterized by specific loss parameters. (*b*) The total optical transmission depicted as a function of propagation distance, normalized by one coupling length, *L_C_*, across various scenarios of optical losses, as determined by the width of the copper film.

In our research, the waveguide configuration depicted in figure 8 was employed in finite-element simulations to study optical transmission as a function of optical loss, defined by the width of the copper film. Figure 9*a* presents the normalized total transmission of the system as a function of *γ*, indicating a minimum total transmission of 0.32 at *γ* = 2.87 mm^−1^, corresponding to *w_f_* of 80 nm. As expected, this minimum transmission value is comparable to 2*μ* for the predefined coupling gap of 1.6 µm.

Practical implementation of this optofluidic *PT*-symmetry system necessitates the coupled waveguide system having at least one coupling length, *L_C_*​. Should there be an intention to integrate this optofluidic *PT* system within a larger system or subsystem, a comprehensive understanding of the influence exerted by the length of the coupled waveguides on various phases of *PT*-symmetry becomes paramount. Such understanding is essential, especially as one may need a more extended coupled waveguide system to accommodate the spatial requirements of a larger system or subsystem. To investigate the impact of waveguide length on the *PT* system, we employed finite-element simulations to observe the total optical transmission as a function of propagation distance, normalized by the coupling length, *L*_C_, for several cases of *γ* defined by *w*_f_. ‘Normalized transmission’ is defined as the ratio of the total power transmitted from both waveguides to the input optical power at the input port. The results of this study are presented in figure 9*b*, revealing that the minimum total optical transmission occurs at an optical loss approximately equivalent to twice the coupling strength and extending up to 4*L_C_*.

For those aiming to use optofluidic *PT*-systems in sensing applications, a comprehensive understanding of a parameter known as the evanescent field ratio (*EFR*) proves to be essential. The *EFR* is defined as the ratio of the evanescent field power within the liquid cladding to the total modal power in the system. This dimensionless quantity serves as a critical determinant in characterizing the interaction between waveguides and the surrounding fluidic environment. The mathematical expression for *EFR* can be formulated as

**Figure 10.**
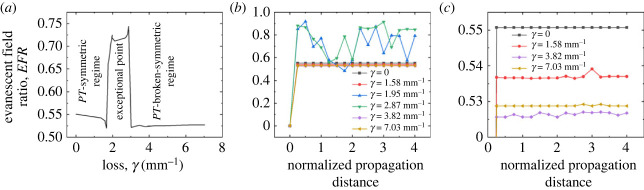
(*a*) Evanescent field ratio of the coupled waveguide system plotted against optical loss, revealing distinct *PT* phases associated with specific loss parameters. (*b*) Evanescent field ratio mapped against the propagation distance, normalized to one coupling length, *L*_C_, for different optical loss scenarios determined by the copper film width. (c) Expanded view of the evanescent field ratio ranging from 52% to 55%, plotted as a function of propagation distance and normalized by one coupling length, *L*_C_.

3.2 $$\;EFR=\frac{\int \int_{\mathrm{Liquid}}\vec{S}\cdot \vec{n}dydz}{\int \int_{All}\vec{S}\cdot \vec{n}dydz},$$

where $\vec{S}$ is the Poynting vector, and $\vec{n}$ is the normal vector to the surface of integration [43]. Contrary to the behaviour of the total optical transmission, the *EFR* exhibits a pronounced enhancement precisely at the juncture where the total optical transmission reaches its nadir, predominantly in proximity to the exceptional point, as delineated in figure 10*a*. In the particular scenario under scrutiny, where a single coupling length is employed, an *EFR* value nearing 0.74 is attained, corresponding to a span of optical loss values that range from 1.95 to 2.87 mm^−1^. This observation elucidates the intricate relationship between the *EFR* and optical loss. It not only illuminates another approach for mapping different *PT* regimes—including *PT*-symmetric, *PT*-broken-symmetric and exceptional point regimes—but also serves as a pivotal consideration for the advancement of chip-scale *PT*-symmetric optical sensing applications.

In alignment with the examination of total optical transmission, as previously studied in this paper, we extend our inquiry to explore the influence of the length of the coupled waveguides on the *EFR*. Utilizing finite-element simulations, we have analysed the *EFR* as a function of propagation distance, normalized by *L*_C_ for several cases of *γ* defined by *w_f_*. The outcomes of this investigation are illustrated in figure 10*b*, where the *EFR* is discerned to range from 50% to 93% across 1 to 4 times the coupling length. More specific *EFR* plots, depicting values between 52% and 55% as a function of propagation distance, are presented in figure 10*c*.

## 4. Conclusion

In conclusion, this research effectively underscores the capabilities of an optofluidic *PT* system in enhancing the evanescent coupling between paired optical waveguides. Within the framework of our investigation, silicon nitride was chosen as the optical waveguide medium, complemented by copper as the optically lossy component. Furthermore, we employed an experimental liquid that exhibits a refractive index 4% lower than that of the core waveguides. While our study focused on specific materials, the expansive arena of optofluidics offers a plethora of material choices. Potential optical waveguides can be crafted from diverse materials like silicon, silicon dioxide, lithium niobate, tantalum pentoxide, titanium dioxide, magnesium fluoride, potassium titanyl phosphate and polydimethylsiloxane. In parallel, metals such as gold, silver, aluminium, titanium, nickel, platinum, palladium, molybdenum, chromium and tungsten can be selected for their optically lossy characteristics, thereby providing a versatile array of optical properties.

We have explored the potential of designing geometries for optically lossy materials tailored for operation within distinct regimes: *PT*-symmetric, *PT*-broken-symmetric and exceptional point. This approach necessitates meticulous design and precise estimation of the loss geometry, which directs the optofluidic system's operation within these specific regimes. A unique feature of our optofluidic platform is its capability to dynamically adjust the refractive index of the liquid cladding in real time. This adjustment is crucial for modulating the optical coupling strength, thereby facilitating the system's transition across different *PT* phases. Specifically, the geometry of the optically lossy material can be initially configured to target the exceptional point. Subsequently, the modulation of the liquid cladding's refractive index enables the non-Hermitian system to transition seamlessly between the *PT*-symmetric, *PT*-broken-symmetric and exceptional point regimes. This versatility underscores the adaptability and precision of our platform in investigating the intricate dynamics of non-Hermitian optofluidic systems.

Future studies in optofluidic passive parity-time-symmetric systems may delve deeper by creating a controlled flow of fluids with periodically varying refractive indices [44] between waveguides. This approach would allow for the periodic modulation of the evanescent coupling between waveguides, thereby facilitating the analysis of effective Floquet Hamiltonians. Furthermore, the method of controlling the flow of fluids with variable refractive indices, used to periodically modulate the evanescent coupling between waveguides, can be extended to manage a flow of microdroplets with varying absorption coefficients between waveguides. Employing this technique could facilitate dynamic encirclement of exceptional points, thereby enabling the capability for optical mode switching.

The comprehensive theoretical modelling, elucidating both the total optical transmission loss and the evanescent field ratios presented in our work, can act as a reference. This foundation paves the way for the design of liquid-enhanced, chip-scale *PT*-symmetric systems, potentially ushering in breakthroughs in light management and advanced sensing applications.

## Data Availability

Data used to generate the plots within this manuscript, along with instructions for their reproduction, are available at the provided Dryad link below. Clicking the link immediately launches a download of the data files only: https://doi.org/10.5061/dryad.bg79cnpgp [45].
